# Epidemiological and clinical assessment of a shared territorial malaria guideline in the 10 years of its implementation (Barcelona, North Metropolitan Area, Catalonia, Spain, 2007–2016)

**DOI:** 10.1186/s12936-017-2007-5

**Published:** 2017-09-11

**Authors:** Josep M. Mòdol, Sílvia Roure, Àlex Smithson, Gema Fernández-Rivas, Anna Esquerrà, Neus Robert, María Méndez, Javier Ramos, Anna Carreres, Lluís Valerio

**Affiliations:** 1 Emergency Department, Hospital Universitari Germans Trias i Pujol, Badalona, Catalonia Spain; 2 Unitat de Salut Internacional Metropolitana Nord, Santa Coloma de Gramenet, Catalonia Spain; 3 Internal Medicine Service, Hospital de l’Esperit Sant, Santa Coloma de Gramenet, Barcelona, Catalonia Spain; 4 Department of Microbiology, Hospital Universitari Germans Trias i Pujol, Badalona, Catalonia Spain; 5 Internal Medicine, Hospital Universitari Germans Trias i Pujol, Badalona, Catalonia Spain; 6 Pediatrics Department, Hospital Universitari Germans Trias i Pujol, Badalona, Catalonia Spain

**Keywords:** Imported malaria, Guideline, *Plasmodium vivax*, Mortality

## Abstract

**Background:**

Malaria remains a major source of morbi-mortality among travellers. In 2007, a consensual multicenter Primary Care-Hospital shared guideline on travel-prior chemoprophylaxis, diagnosis and clinical management of imported malaria was set up in the Barcelona North Metropolitan area. The aim of the study is to assess the evolution of malaria cases in the area as well as its clinical management over the 10 years of its implementation.

**Results:**

A total of 190 malaria cases, all them imported, have been recorded. The overall estimated malaria crude incidence was of 0.47 cases per 10,000 population/year (95% CI 0.34–0.59) with a slight significant positive slope especially at the expense of an increase in Indian sub-continent *Plasmodium vivax* cases. The number of patients who attended the pre-travel consultation was low (13.7%) as well as those with prescribed chemoprophylaxis (10%). Severe malaria was diagnosed in 34 (17.9%) patients and ICU admittance was required in 2.6% of them. Organ sequelae (two renal failures and one post-acute distress respiratory syndrome) were recorded in 3 patients at hospital discharge, although all three were recovered at 30 days. None of the patients died. Patients complying with severity criteria were significantly males (p = 0.04), came from Africa (p = 0.02), were mainly non-immigrant travellers (p = 0.01) and were attended in a hospital setting (p < 0.001). The most frequently identified species was *Plasmodium falciparum* (64.2%), *P. vivax* (23.2%), *Plasmodium malariae* (1.6%) and *Plasmodium ovale* (1.1%). Those patients diagnosed with *P. falciparum* malaria came more often from sub-Saharan Africa (p < 0.001) and those with *P. vivax* came largely from the Indian sub-continent (p = 0.003). Among the 126 patients in whom an immunochromatographic antigenic test was performed, the result was interpreted as falsely negative in 12.1% of them. False negative results can be related to cases with <1% parasitaemia.

**Conclusions:**

After 10 years of surveillance, a moderate increase in malaria incidence was observed, mostly *P. vivax* cases imported from the Indian sub-continent. Although severe malaria cases have been frequently reported, none of the patients died and organ sequelae were rare. Conceivably, the participation of the Primary Care and the District and Third Level Hospital professionals defining surveillance, diagnostic tests, referral criteria and clinical management can be considered a useful tool to minimize malaria morbi-mortality.

## Background

Nowadays malaria can be considered as a non-spreading disease—even as an infection of declining-incidence infection—in most of the countries classically regarded as highly endemic. Nevertheless, this falling number of cases has been not so evident in the European Union (UE) countries where imported malaria remains a major source of morbi-mortality among travelers [1], especially in areas where malaria is less regularly seen and treated [2]. Various reasons have been suggested to explain this: longer stays in high-transmission areas, increasing number of visiting friends and relatives (VFRs) travellers or the changing patterns of business journeys after the 2008 UE economic crisis [3]. Whatever the cause, the availability of clear, simple and WHO-based guidelines on diagnosis and treatment of malaria—always adapted to local realities—is a key-point when intending to achieve clinical management excellence. Since the constitution of the Barcelona North Metropolitan International Health Programme (PROSICS MetroN) in 2007, a consensual and yearly-updated set of guidelines on travel-prior chemoprophylaxis, diagnosis and clinical management of imported malaria cases has been accessible in every health facility of that health area. The main aim of the study is to assess the evolution of malaria cases in the area as well as its clinical management and in the frame of the malaria guideline over the 10 years of its implementation.

## Methods

**Fig. 1:**
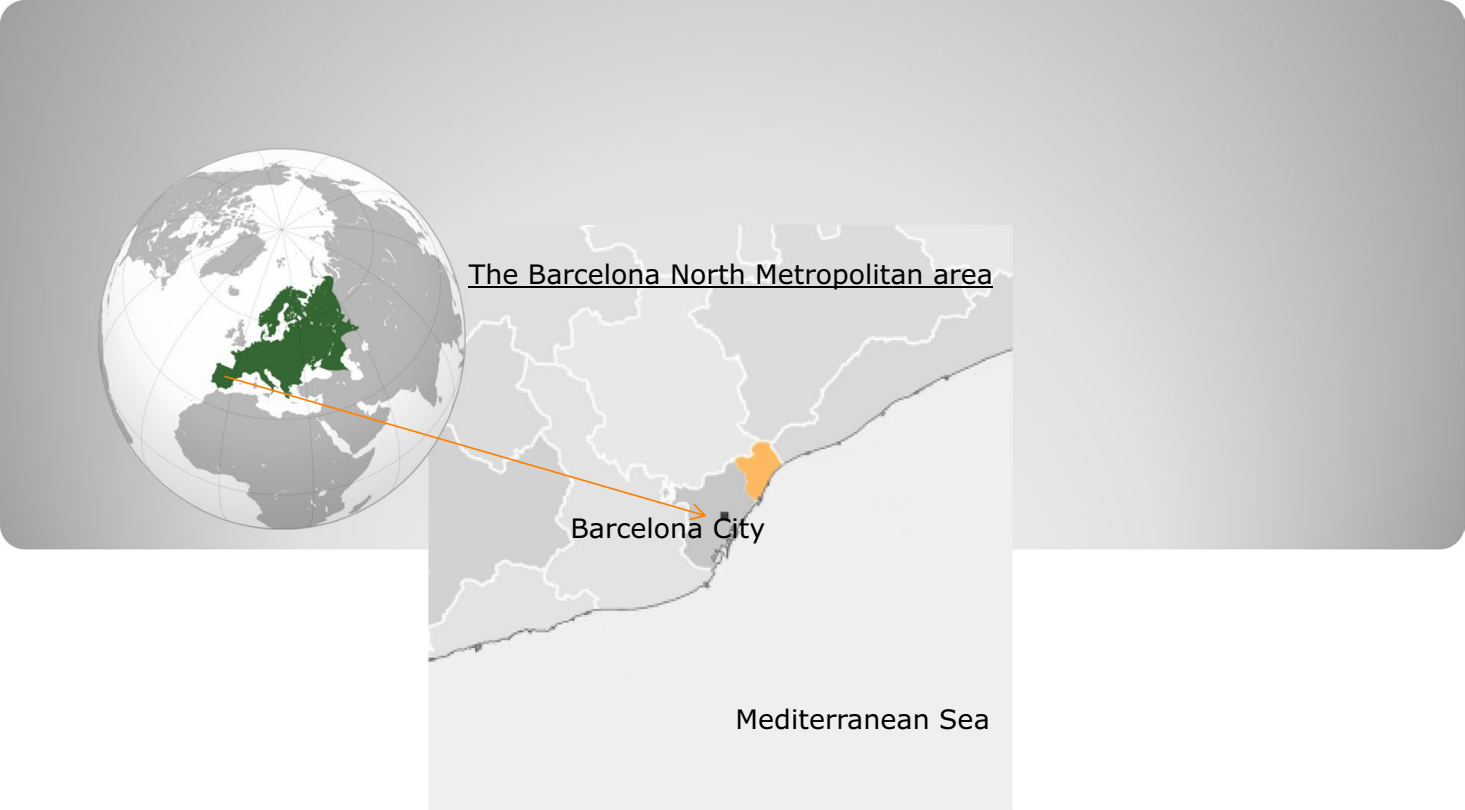
The geographic area of study

After the first approval and implementation of the shared malaria guideline (in 2007), an observational, prospective and multicentre surveillance study has been performed in all the health facilities—located at either hospital or primary health levels—with diagnostic capability in the North Metropolitan Area of Barcelona (Catalonia, Spain) during the period January 2007 to December 2016. Sentinel clinicians (infectious diseases, emergencies and microbiology staff, paediatricians and general practitioners) from the PROSICS MetroN study group voluntarily recorded all consecutive individuals with confirmed malaria at four care levels: (1) one teaching third-level hospital (Hospital Universitari Germans Trias i Pujol; Badalona), (2) one International Health Unit (Unitat de Salut Internacional Metropolitana Nord; Santa Coloma de Gramenet), (3) one district hospital (Hospital de L’Esperit Sant; Santa Coloma de Gramenet) and, (4) the territorial 22 Primary Care centers (see Fig. 1). The overall population average of the Barcelona North Metropolitan area along the study period was of 412,000 inhabitants; with stable proportions of immigrants from any origin ranging from 16.9% (2007) to 15.8% (2016) largely from malaria endemic countries (mean proportions of the study period: Indostani immigrants = 3.6%, sub-Saharan immigrants = 2.7%).

All these health facilities belong to the public Catalan Health Service in the frame of the PROSICS and, therefore, the medical visits are easily accessible and free of charge. Likewise, the territorial International Health Unit (conducting pre-travel preventive activities and being by far the main responsible of anti-malarial chemoprophylactic prescriptions) also belongs to the same public provider. Individuals were considered as infected cases when any clinical suspicion of acute disease defined as the presence of antecedents of travelling to endemic areas and a clinical compatible presentation was confirmed by means of one positive thick or thin blood film. In most of the cases, in addition, an antigenic immunochromatography test (SD Bioline Malaria Ag Pf/Pv©, Standard Diagnostics Inc, Germany) was performed. Diagnostics based on a high clinical suspicion, but with negative tests and patients residing outside the Health Area were excluded from the study.

The variables assessed were: age, sex, immigrant (yes/no), risk factors for severe malaria, travel destination, trip length (days), reason for travel (tourism, VFR, business, other), prior travel advise (yes/no), chemoprophylaxis (yes/no), chemoprophylactic drug (chloroquine, mefloquine, chloroquine + proguanil, atovaquone + proguanil) or stand-by treatment (atovaquone + proguanil), diagnostic method (blood film, PCR, immunochromatography), *Plasmodium* species (*Plasmodium falciparum, Plasmodium vivax, Plasmodium ovale, Plasmodium malariae, Plasmodium knowlesi, Plasmodium* spp. and coinfection), parasitaemia percentage, severity criteria (yes/no), care level (outpatient, Emergencies Short Stay area, Infectious Diseases ward, ICU), treatment, clinical evolution and outcome.

Every person born outside the European Union (EU) was considered as immigrant. Immigrants with imported malaria epidemiologically related with a recent travel to their native country after residing for >2 years in the EU were considered as Visiting Friends and Relatives (VFRs). The major risk factors for malaria were pregnancy, age < 5 years and immunodeficiency. When assessing travel destination, a categorization according biogeographic areas was used instead of countries visited, as follows: sub-Saharan Africa, America and the Indian sub-continent (no cases have been recorded from South–East Asia or Oceania). Parasitaemia percentage refers the proportion (%) of erythrocytes with visible asexuals forms of *Plasmodium* in thin blood film; proportions of <0.5% were assumed to this value. A malaria case was considered as severe when a least one WHO criteria was identified: Glasgow (adults) <11 or Blantyre (children) coma scores) <3, Hb <5 g/dL, creatinine >3 mmol/L, pH < 7.25, hypoglycaemia <2.2 mmol/L, jaundice or bilirubin >3 mg/dL, oxygen saturation <92% or radiologically-compatible signs of APO, haemoglobinuria, spontaneous bleeding (excluding episodic epistaxis) or disseminated intravascular coagulation (DIC) criteria, seizures, shock (<70 or 80 mmHg in systolic blood pressure, children and adults respectively) and parasitaemia >4%. Uncomplicated malaria cases were treated whether with atovaquone + proguanil or chloroquine depending on the species and the resistance pattern. When *P. vivax/P. ovale* was identified, patients received a subsequent treatment with primaquine phosphate 30 mg/kg per 14 days. Severe malaria cases were treated with quinine i.v. plus doxycycline according to the WHO-based guideline; since mid 2013 artesunate was used instead.

The relative frequency of the variables and their association with socio-demographic characteristics were analysed using SPSS 19.0 software (SPSS Inc, Chicago, IL). The Chi square test, with Fisher’s correction if needed, was used to compare qualitative variables. The p value was set at 0.05 for statistical significance. In order to evaluate the evolution of the disease, two time periods were defined: period 1 (2007–2011) and period 2 (2012–2016). In this line, the trend in the incidence of malaria during the study period was estimated by defining its trendline (a linear equation), which was calculated according to a model *y* = *mx* + *b*; in which y = number of cases and x = time (years); *m* can be deduced from the Least Squares Method and represents the fact that the difference in the *y* coordinate between two points on a line (that is, *y* − *y*_1_) is proportional to the difference in the *x* coordinate (that is, *x* − *x*_1_). In short, the proportionality constant *m* represents the slope of the trendline and *b* the point where the graph crosses the *y*-axis. Furthermore, a tendency test (IRR: incidence relative risk) was performed by Poisson Regression.

Authorization for the execution of the study was obtained after evaluation by the Ethical Review Boards of the Primary Health Area, both hospitals and the Public Health Service (PI-17-047).

## Results

A total of 190 malaria cases, all them imported, have been recorded in 187 patients (one patient having two different episodes and another one, three. No relapses were recorded); 164 (87.8%) out of those 187 were immigrants. Up to 146 (76.8%) immigrants were considered as VFRs while 18 (9.5%) malaria cases were diagnosed among newly arrived immigrants. Thus, the overall estimated malaria crude incidence was of 0.47 cases per 10,000 population/year (95% CI 0.34–0.59). Among immigrants, that ratio showed an incidence of 2.5 cases per 10,000 population/year (95% CI 2.21–2.80).

On the whole, the number of patients who attended the pre-travel consultation was low (26 cases; 13.7%) as well as those with prescribed chemoprophylaxis (19 cases; 10%). All those 19 patients with malaria in spite of a proper chemoprophylaxis prescription came from sub-Saharan Africa; 17 (89.5%) of them abandoned the treatment during the travel (mefloquine: 10, atovaquone + proguanil: 3 and doxycycline: 4) and 2 were considered as real chemoprophylactic failures (both involving mefloquine).

**Fig. 2:**
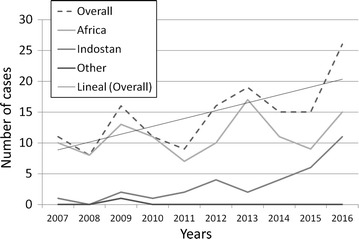
Number of malaria cases (2007–2016)

Socio-demographic and clinical data with a further bivariate analysis in relation to the presence of severity criteria and the malaria casuistic comparing two 5-years long periods are shown in Tables 1 and 2. Patients complying with severity criteria were significantly males (p = 0.04), coming from Africa (p = 0.02), were mainly non-immigrant travellers (p = 0.01) and were attended in a hospital setting (p < 0.001). Patients from Africa tended to consult more, in first instance to the Primary Care or International Health external services (p < 0.001), while Indostani patients were attended mainly in hospital devices (p < 0.001). The overall malaria incidence during the study period has displayed a slight significant positive slope (y/x = 1.28; IRR = 1.1, 95% IC 1.05–1.18), especially at the expense of an increase in Indostanic *P. vivax* cases though none of both African or Indostani linear trend reach significance when analysed separately as shown in Fig. 2.

**Table 1:** Epidemiological and clinical analysis according to severity

	Total (%)	Severe malaria (%)	Uncomplicated malaria (%)	p
Number of cases	190 (100)	34 (17.9%)	156 (82.1%)	–
Mean age (DE)	32 (13.8)	35.3 (11.1)	31.2 (14.3)	NS
Sex				
Men	121 (63.7)	27 (79.4)	94 (60.3)	0.04
Women	69 (36.3)	7 (20.6)	62 (39.7)	
Species				
*P. falciparum*	122 (64.2)	27 (79.4)	95 (60.9)	NS
*P. vivax*	44(23.2)	4 (11.8)	40 (25.6)	
*P. ovale*	2 (1.1)	0 (0)	2 (1.3)	
*P. malariae*	3 (1.6)	0 (0)	3 (1.9)	
*P.* spp.	14 (7.4)	2 (5.9)	12 (7.7)	
Co-infections^a^	5 (2.6)	1 (2.9)	4 (2.6)	
Positive Immunocromatography (N = 124)	109 (87.9)	27 (90)	82 (85.4)	NS
Travel destination				
Sub-Saharan Africa	142 (74.7)	33 (97.1)	109 (69.9)	0.02
Indian sub-continent	46 (24.2)	1 (2.9)	45 (28.8)	0.003
Amèrica	2 (1.1)	0 (0)	2 (1.3)	
Length of trip ≤ 30 days (N = 147)	39 (26.5)	10 (29.4)	29 (18.5)	NS
Presence of risk factors^b^	8 (4.2)	0 (0)	8 (5.1%)	NS
Immigrant	164 (86.3)	25 (73.5)	139 (89.1)	0.01
Travel reason				
Tourism	8 (4.2)	5 (14.7)	3 (1.9)	
VFRs	171 (90)	25 (73.5)	146 (93.6)	<0.001
Business	11 (5.8)	4 (11.8)	7 (4.5)	
Prior travel advice	26 (13.7)	5 (14.7)	21 (13.4)	NS
Chemoprophylaxis	19 (10)	4 (11.8)	15 (9.6)	NS
Care level				
Primary care	64 (33.7)	2 (5.9)	62 (39.7)	<0.001
Emergency area	34 (17.9)	3 (8.8)	31 (19.9)	
Medicine ward	87 (45.8)	24 (70.6)	63 (40.4)	<0.001
ICU	5 (2.6)	5 (14.7)	0 (0)	
Treatment				
Quinine	71 (37.4)	29 (85.3)	42 (26.9)	<0.001
Artemether	4 (2.1)	1 (2.9)	3 (1.9)	
Atovaquone/Prog.	62 (32.6)	2 (5.9)	60 (38.5)	<0.001
Other	53 (27.9)	2 (5.9)	51 (32.7)	
Sequelae at discharge	3 (1.6)	3 (8.8)	0 (0)	NS
Favourable clinical evolution (to 30 days)	190 (100)	34 (100)	156 (100)	–
Mortality	0 (0)	0 (0)	0 (0)	–

^a^ *P. falciparum*-*P. malariae*: 1, *P. falciparum*-*P. ovale*: 4

^b^Immunodeficiency: 5, pregnancy: 2, age < 1: 1

**Table 2:** Epidemiological and clinical analysis according to 5-years periods

	Total (%)	Period I (2007–2011) (%)	Period II (2012–2016)^a^ (%)	p
Number of cases	190 (100)	72 (37.9)	118 (62.1)	–
Incidence (/10,000 hab/year)	0.47	0.36	0.58	<0.001
Mean age (DE)	32.0 (13.8)	30.3 (12.5)	33 (14.5)	NS
Sex				
Men	121 (63.7)	50 (69.4)	71 (60.2)	NS
Women	69 (36.3)	22 (30.6)	47 (39.8)	
Species				
*P. falciparum*	122 (64.2)	55 (76.4)	67 (56.8)	
*P. vivax*	44 (23.2)	6 (8.3)	38 (32.2)	<0.001
*P. ovale*	2 (1.1)	2 (2.8)	0 (0)	
*P. malariae*	3 (1.6)	2 (2.8)	1 (0.8)	
*P.* spp.	14 (7.4)	5 (6.9)	9 (7.6)	
Co-infections^b^	5 (2.6)	2 (2.8)	3 (2.5)	
Positive immunocromatography (n = 126)	109 (86.5)	40 (88.9)	69 (85.2)	NS
Travel destination				
Sub-Saharan Africa	142 (74.7)	66 (91.7)	76 (64.4)	<0.001
Indian sub-continent	46 (24.2)	6 (8.3)	40 (33.9)	<0.001
America	2 (1.1)	0 (0)	2 (1.7)	
Length of trip ≤ 30 days (n = 147)	39 (26.5)	19/54 (35.2)	20/93 (21.5)	0.07
Presence of risk factors^c^	8 (4.2)	4 (5.6)	4 (3.4)	NS
Immigrant	164 (84.3)	63 (87.5)	101 (85.6)	NS
Travel reason				
Tourism	8 (4.2)	5 (6.9)	3 (2.5)	0.04
VFR	171 (90)	66 (91.7)	105 (89)	
Business	11 (5.8)	1 (1.4)	10 (8.5)	
Prior travel advice	26 (13.7)	6 (8.3)	20 (16.9)	NS
Chemoprophylaxis	19 (10)	4 (5.6)	15 (12.7)	NS
Severity	34 (17.9)	13 (18.1)	21 (17.8)	NS
Parasitaemia > 4%	19 (10)	7 (9.7)	12 (10.2)	NS
Treatment				
Quinine	71 (37.4)	37 (51,4)	34 (28.8)	0.001
Artemether	4 (2.1)	0 (0)	4 (2.5)	
Atovaquone/Prog.	62 (32.6)	17 (23.6)	45 (38.1)	0.003
Other	53 (27.9)	17 (23.6)	36 (30.5)	
Sequelae at discharge	3 (1.6)	0(0)	3 (2.5)	NS
Favourable clinical evolution (to 30 days)	190 (100)	72 (100)	118 (100)	NS
Mortality	0 (0)	0 (0)	0 (0)	NS

^a^Artemisinin-available period

^b^ *P. falciparum*-*P. malariae*: 1, *P. falciparum*-*P. ovale*: 4

^c^Immunodeficiency: 5, pregnancy: 2, age < 1: 1

The most frequently identified species was *P. falciparum* (122 cases; 64.2%) and, subsequently, *P. vivax* (44 cases; 23.2%), *P. malariae* (3; 1.6%) and *P. ovale* (2 cases; 1.1%). Co-infection was recognized in 5 (2.6%) cases: 1 *P. falciparum* + *P. malariae* and 4 *P. falciparum* + *P. ovale*. Forteen cases (7.4%) were labeled as *Plasmodium* spp. Those patients diagnosed of *P. falciparum* malaria came more often from Sub-Saharan Africa (p < 0.001) and those with *P. vivax* came largely from the Indian sub-continent (p = 0.003). Among the 124 patients in which an immunochromatographic antigenic test was performed, the result was interpreted as falsely negative in 15 (12.1%) of them. False negative results can be related to lower <1% parasitaemia but no relation with the specific infecting specie was found. The distribution of cases according to identified species, parasitaemia degree and the performance of the antigenic test are shown in Table 3.

**Table 3:** Parasitaemia (%) according to species and performance of the antigen-immunochromatographic test (n = 124)

Parasitaemia	<1%	1-4%	>4%	Total
*P. falciparum* ^a^				
Antigenic rapid test+ (%)	35 (81.4)	40 (95.2)	6 (100)	81 (89.2)
Antigenic rapid test− (%)	8 (19.6)	2 (4.8)	0 (0)	10 (10.8)
*P. vivax*				
Antigenic rapid test + (%)	13 (76.5)	14 (93.3)	1 (100)	28 (84.8)
Antigenic rapid test− (%)	4 (24.4)	1 (6.7)	0 (0)	5 (15.2)
Total				
Antigenic rapid test+ (%)	48 (80)	54 (94.7)	7 (100)	109 (87.9)
Antigenic rapid test− (%)	12 (20)^b^	3 (6.3)	0 (0)	15 (12.1)

^a^Co-infections: *P. falciparum* + *P. ovale* = 3 cases 1–4%, 1 case > 4%; *P. falciparum* + *P. malariae* = 1 case 1–4%

^b^p = 0.01 when comparing number of <1% false negatives (20.2%) vs ≥1% (6.7%)

During the 10 years-long study period, up to 127 malaria episodes (66.8%) in 125 patients were hospitalized and severe malaria was diagnosed in 34 (17.9%) patients. In order of frequency, the most recorded criteria of severity were hyperparasitaemia (19 patients), DIC (7), hyperbilirubinaemia (4), APO (3), Hypercreatinaemia (2), seizures (1) and low Glasgow score (1); three patients having two concurrent criteria. ICU admittance was required in 5 (2.6%) of them. Organ sequelae (2 renal failures and one post-acute distress respiratory syndrome) were recorded in 3 patients at hospital discharge, although all three were recovered at 30 days. None patient died.

## Discussion

Different quantitative and qualitative aspects have to be taken into consideration when addressing the impact of imported malaria. With regard to quantitative relevance of the disease, surprisingly, there are very few studies estimating incidences of malaria in European countries and, moreover, they diverge considerably because of the difficulty of knowing the denominators. In the whole, the study show malaria incidences tending to be more in line with those of the two major importing-countries (France and the UK), both stating around 2000 cases/year, than those observed in Spain (with over 300–500 cases/year) [4]. Some degree of official under-reporting in Spain could be postulated in the same way as others authors have warned for their own countries [5]. The slight though significant arise of the disease during the ten-years study period has concurred with the increase of *P. vivax* and, in a less number, of *P. falciparum* cases coming from Pakistan from 2012 onwards in an area where Indostani immigrants are the larger immigrant community. A similar pattern has been described in other European countries [6, 7] in contrast with the Spanish global data (4.2% of *Plasmodium* identifications) [8]. This aspect has relation with the qualitative assessment of the malaria casuistic in Southern European regions such as Catalonia: limited outbreaks of *P. vivax* malaria have taken place in areas ecologically similar [9] (i.e.: The Peloponnesus, Corsica), after unrecorded importations. More and better resources should be allocated to health and entomological surveillance programmes. Although the global risk of re-introduction of malaria in Spain can be considered as low [10], the reality points to an increasing number of imported cases declared from Mediterranean countries (Albania, Greece, Israel and, especially, Italy) with a very scarce recent historical record [11].

Likewise in other European large studies, the archetype of patient with imported malaria corresponds to a VFRs young immigrant [12]. Nevertheless, up to a 24.2% of cases came from Asia—all them from the Indian subcontinent—with *P. vivax* accounting for a 23.2% of parasite identifications. Of course, this fact can be explained by the immigrant background of the Barcelona North Metropolitan area, but, in spite of this, the results suggest that current policies should undergo a strategic change. The widely-accepted low risk of importing malaria from the Indian sub-continent may be reassessed as the meta-analysis of Tatem et al. suggests [13]. Along with others, the authors support actions such as addressing malaria guidelines, spreading pre-travel information among immigrants in the frame of a community approach or improving the diagnostic capacities of general practitioners and emergency areas [14].

Patients stating previous travel advice accounted just a 13.7% of cases. Among patients in whom some chemoprophylactic drug was prescribed there were very few documented real drug failures, the wide majority of them having not been fully compliant. These data advocate that correct drug prophylaxis can effectively prevent malaria cases. Counseling of travellers on malaria prevention should be improved and coverage extended [15].

An integrated assistance model allows malaria cases to be treated at different levels of specialization without causing greater morbidity than that described in other studies. Such an efficient model was based in a net of trained general physicians able to distinguish between mild and severe malaria cases always with a close, quick and continuous support of an International Health Unit with full-time specialized personnel. Recent immigrants from sub-Saharan Africa most of them VFRs partially semi-immune to malaria and with lower risk of death (a principle not applicable, however, to long-term African immigrants) [16], take advantage of this work organization reducing hospital admittances and costs. That said, the model presents weaknesses when assessing the diagnostic possibilities in primary care: consistently with many other studies, immunochromatographic tests lack utility among patients with low parasitic loads such as a most part of malaria cases attended in primary care not to mention his inadequacy against *P. malariae* and *P. ovale*. To date, thick and thin blood films (or PCR when and where possible) are irreplaceable, and make it necessary to endow primary care centres with specific care-points and expert personnel. During the ten years-period, two of these care-points were fully accessible, located at the International Health Unit and in the third level Emergency Area hospital. After the analysis of data, the use of immunochromatography has been abandoned.

A similar inter-level model could be especially adequate in areas with presence of a large proportion of African VFRs travellers, a population with little proclivity to pre-travel advice and generally reduced risk of severe malaria [17]. In a recent article performed in East London the authors conclude doctors must familiarize themselves with ambulatory management of malaria since only an 8.5% were treated as outpatients [18]. Probably hospital physicians should step out the hospital and adopt a closer position nearer the community not in competitive but in collaborative terms with Primary Care physicians to mutual and patients benefice.

The study has showed one of the lowest mortality rates (none death) ever recorded in Europe despite the fact that the percentage of severe malaria cases was 17.9%, a proportion which is close or slightly above what has been previously published [19]. This result may support that the knowledge and management of the disease according to the guideline by both the Primary Care and hospital physicians is in the whole adequate. The presence of complications was also low. Although semi-immune individuals have a considerably lesser risk to progression to severe malaria, two additional facts suggest the model has a rapid and well-organized reaction capability: the small number of patients requiring ICU admittance (5) and the absence of early severe complications, such as cerebral malaria. Renal failure and post-malarial respiratory distress appear usually in the middle or later stages of the disease [20]. Interestingly, in the part of the study prior to the artesunate introduction (2013) there were also no deaths. This would suggest that an adequate organization is at least as important as an easy access to the more effective drug-based treatment.

One of the main study weaknesses concerns the relatively high number of *Plasmodium* unidentified species (7.4%), particularly at primary care level. Possibly the guideline focuses more on organizational issues than on microbiological ones, an aspect to be addressed in future updates.

## Conclusions

After 10 years of surveillance, malaria remains a relevant health problem with an even moderate increase at the expense of *P. vivax* cases imported from the Indian sub-continent. In areas with substantial numbers of VRF immigrants, a proactive attitude and the use of updated consensual specific guidelines with the participation of primary care, district and third level hospital professionals defining surveillance, diagnostic tests, referral criteria and clinical management could be a useful tool to minimize severe cases and mortality.
